# Fixing PAN Nanofiber Mats during Stabilization for Carbonization and Creating Novel Metal/Carbon Composites

**DOI:** 10.3390/polym10070735

**Published:** 2018-07-04

**Authors:** Lilia Sabantina, Miguel Ángel Rodríguez-Cano, Michaela Klöcker, Francisco José García-Mateos, Juan José Ternero-Hidalgo, Al Mamun, Friederike Beermann, Mona Schwakenberg, Anna-Lena Voigt, José Rodríguez-Mirasol, Tomás Cordero, Andrea Ehrmann

**Affiliations:** 1 Faculty of Engineering and Mathematics, Bielefeld University of Applied Sciences, 33619 Bielefeld, Germany; lilia.sabantina@fh-bielefeld.de (L.S.); michaela.kloecker@fh-bielefeld.de (M.K.); friederike.beermann@fh-bielefeld.de (F.B.); mona.schwakenberg@fh-bielefeld.de (M.S.); anna-lena.voigt@fh-bielefeld.de (A.-L.V.); 2 Departamento de Ingeniería Química, Campus de Teatinos s/n, Universidad de Málaga, Andalucía Tech, 29010 Málaga, Spain; marodriguez@uma.es (M.Á.R.-C.); garciamateos@uma.es (F.J.G.-M.); ternerohidalgo@uma.es (J.J.T.-H.); mirasol@uma.es (J.R.-M.); cordero@uma.es (T.C.); 3 Faculty of Textile and Clothing Technology, Niederrhein University of Applied Sciences, 41065 Mönchengladbach, Germany; mamun26th@gmail.com

**Keywords:** polyacrylonitrile, PAN, nanofiber mat, electrospinning, stabilization, dimension stability, carbonization

## Abstract

Polyacrylonitrile (PAN) is one of the materials most often used for carbonization. PAN nanofiber mats, created by electrospinning, are an especially interesting source to gain carbon nanofibers. A well-known problem in this process is fixing the PAN nanofiber mats during the stabilization process which is necessary to avoid contraction of the fibers, correlated with an undesired increase in the diameter and undesired bending. Fixing this issue typically results in breaks in the nanofiber mats if the tension is too high, or it is not strong enough to keep the fibers as straight as in the original state. This article suggests a novel method to overcome this problem by electrospinning on an aluminum substrate on which the nanofiber mat adheres rigidly, stabilizing the composite and carbonizing afterwards either with or without the aluminum substrate to gain either a pure carbon nanofiber mat or a metal/carbon composite.

## 1. Introduction

Electrospinning can be used to create nanofiber, e.g., from polyacrylonitrile (PAN) which is a typical precursor of carbon nanofibers [1,2,3]. Such carbon nanofibers can be applied, e.g., to improve the mechanical properties of plastic materials by forming a composite with a polymer or a resin, the electrical properties of batteries and super-capacitors, etc. [4,5,6,7].

To gain carbon nanofibers, the first step is a stabilization process which is typically performed in air, resulting in cyclization and thermally stable aromatic ladder polymer formation [8] which increases the chemical and mechanical stability of the nanofiber mat and is essential before the carbonization step. The whole stabilization process also includes dehydrogenation, aromatization, oxidation, and crosslinking [9,10,11].

In previous articles discussing the stabilization and subsequent carbonization of PAN nanofiber mats, stabilization temperatures, and heating rates are strongly discussed. Fitzer et al., e.g., investigated different stabilization temperatures between 260 and 290 °C and heating rates up to 5 °C/min, showing that for the PAN they used a heating rate of 1 °C/min and a final temperature of 270 °C resulted in maximum tensile strength of the final carbon fibers carbonized at 1350 °C [12]. On the contrary, Mathur et al. found that temperatures up to 300 °C were not sufficient for thermal stabilization, but temperatures between 350 and 400 °C were needed to reach low hydrogen contents and correspondingly only little tar formation during carbonization [13]. A completely different thermal treatment was found ideal by Moon and Ferris. They suggested performing a first stabilization step with a high heating rate of 5 °C/min up to 150 °C, followed by a first isothermal treatment for 2 h to completely remove water and solvent. Afterwards, stabilization was carried further to 200 °C where a second isothermal step followed for 2 h. Carbonization was even split in four steps with decreasing heating rates up to a temperature of 1350 °C. In this way, the best ultimate strengths of the carbon yarns were reached [14]. Mólnar et al. found different conversion temperatures for the completed stabilization process, depending on the measurement method they applied. The color examination revealed the lowest conversion temperature of approx. 195 °C, followed by the temperature based on FTIR of 207 °C, while the DSSC indicated a conversion temperature of 244 °C. In all cases, the standard deviations were found to be 34–56 °C; thus these conversion temperatures are not exact values [15]. Gu et al. examined the conductivity of carbon nanofibers, stabilized at different temperatures, and found that while the morphology of the carbon nanofibers was desirable for a stabilization temperature of 250 °C, a stabilization temperature of 270 °C resulted in fiber conglutinations which were supportive for an increased conductivity [16]. Rafiei et al. found stabilization temperatures of 150–270 °C in combination with a heating rate below 2 °C/min ideal according to the stabilization index and the aromatization index [17]. 

A problem which is less often mentioned, however, is the dimensional change of the nanofibers during the stabilization process. On the one hand, conglutinations are formed which are sometimes helpful [16] but in most cases undesired. Some publications show such conglutinations in the SEM images without discussing them further [18,19] or describing how to overcome this problem by changing solution and stabilization parameters [20]. On the other hand, shrinkage and bending of fibers may occur if they are not fixed during the temperature treatment. Ma et al. stretched a bundle of PAN nanofibers by knotting them together with a carbon fiber cord, tying them with a metal hook, and tying the other cord with a displacement device to apply a programmed tension, in this way stretching them by approximately a factor of 3 and afterwards stabilizing them at fixed length by stretching them over a metal frame [21]. Wu et al. used hot-stretching during stabilization to gain an elongation by a factor of 1.7, fixing one side of the sample on a frame and putting a defined weight at the opposite side [22]. Xie et al. stretched the fibers in an oven by applying a weight at a middle temperature of 140 °C up to different pre-defined drawing ratios, followed by stabilization at 250 °C for 4 h under a constant load. Comparison between raw and drawn yarn showed an increase in yarn and fiber uniformity after drawing as well as better fiber alignment, polymer chain orientation, and corresponding tensile strength [23]. Ma et al. found that even the tension during carbonization influenced the tensile strength and Young’s modulus, suggesting a moderate carbonization tension of 20 cN per nanofiber bundle [24]. 

These experiments are usually applied to nanofiber bundles and cannot be transferred to nanofiber mats produced with needleless electrospinning. Such samples are either stabilized freely or fixed during this process to avoid conglutinations and undesired morphological changes of the fibers. Fixing the samples, however, is not easy. If only two opposite sides are fixed by a weight, e.g., the other sides will shrink [25,26]. If all sides are fixed, the samples can break even at low heating rates since the forces working on the nanofiber mat are not well distributed [27,28].

This article thus aims at suggesting a simple new approach to overcome this problem which at the same time offers the possibility to create a novel metal–carbon composite. It should be mentioned that the mechanical properties of the single nanofibers, as well as the nanofiber mats, were not investigated. The first is technologically quite demanding; the latter does not correspond to the planned application of single carbon nanofibers in composites. Instead, the focus of the recent study is the development of an increased stabilization method as well as a new method to create metal/carbon composites.

## 2. Materials and Methods

The electrospinning machine—Nanospider Lab (Elmarco, Liberec, Czech Republic), a needleless electrospinning machine based on the wire technology—was used to create nanofiber mats. Spinning parameters were as follows: high voltage 60 kV, current approx. 0.04 mA, electrode–substrate distance 240 mm, nozzle diameter 0.8 mm, carriage speed 50 mm/s, substrate speed 50 mm/min, relative humidity 33%, and temperature 22.0 °C.

The spinning solution contained 15% PAN dissolved in DMSO (min 99.9%, purchased from S3 chemicals, Bad Oeynhausen, Germany).

As a substrate, household aluminum foil (from Rewe, Bielefeld, Germany) was used. For comparison, samples electrospun on the usual polypropylene (PP) substrate (from Elmarco) under identical conditions are used.

Samples of the electrospun nanofiber mats were stabilized in a muffle furnace B150 (Nabertherm, Lilienthal, Germany), approaching a typical stabilization temperature of 280 °C at a heating rate of 1 °C/min, followed by isothermal treatment at this maximum temperature for 1 h. The samples electrospun on PP were separated from the substrates before stabilization (since PP melts below stabilization temperature), while the samples electrospun on aluminum were not separated from their substrates. For carbonization, a furnace CTF 12/TZF 12 (Carbolite Gero Ltd., Hope, UK) was used, approaching temperatures of 500 °C or 800 °C, respectively, with a heating rate of 10 °C/min in a nitrogen flow of 150 mL/min (STP), followed by isothermal treatment for 1 h.

Scanning electron microscopy (SEM) images were taken by a Zeiss 1450VPSE (Oberkochen, Germany) with a resolution of 5 nm, using a nominal magnification of 5000×. Additionally, a confocal laser scanning microscope (CLSM) VK-9700 by Keyence (Neu-Isenburg, Germany) was applied. For Fourier-transform infrared (FTIR) spectroscopy, an Excalibur 3100 (Varian, Inc., Palo Alto, CA, USA) was used. The software ImageJ 1.51j8 (from National Institutes of Health, Bethesda, MD, USA) was applied to determine the nanofiber diameters from 50 fibers per sample.

## 3. Results

**Figure 1:**
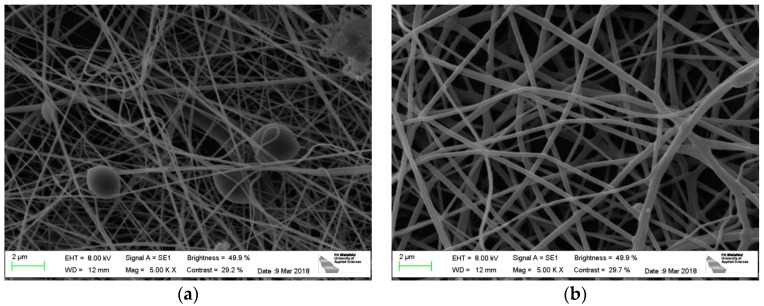
Electrospun PAN nanofiber mats: (**a**) PP as substrate; (**b**) aluminum foil as substrate.

Figure 1 depicts PAN nanofiber mats, prepared during the same electrospinning process, on PP and aluminum foil as substrates, respectively. On the aluminum foil, the nanofibers show a slightly larger diameter, connected with fewer undesired beads. The latter are typically for PAN nanofiber mats spun from DMSO with relatively low solid contents. Apparently, spinning on aluminum foil can on the one hand reduce the beads, probably due to shaping the electric field in the spinning chamber differently; on the other hand, the slightly thicker nanofibers may be unwanted. This shows that spinning and solution parameters must be carefully adjusted to gain the desired morphology if the substrate is changed. This parameter study will be performed in the near future.

**Figure 2:**
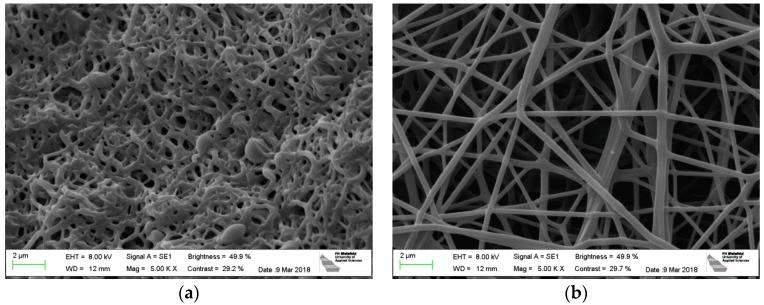
PAN nanofiber mats after stabilization: (**a**) electrospun on PP (substrate separated before stabilization); (**b**) electrospun on aluminum foil (substrate not removed before stabilization).

Figure 2 shows the effect of stabilization on PAN nanofiber mats on both substrates. The nanofiber mat produced on the usual PP substrate and stabilized purely (without the substrate) without fixing it has strong conglutinations between the single fibers, the latter are clearly thicker than in the original state. It should be mentioned that fixing the sample by weights significantly reduces the effect, but cannot completely eliminate it [27,28].

The samples stabilized via adhering on the aluminum foil, however, have not changed their morphology (Figure 2b). Apparently, this simple method can help increasing the formation of long, straight stabilized PAN fibers. 

**Figure 3:**
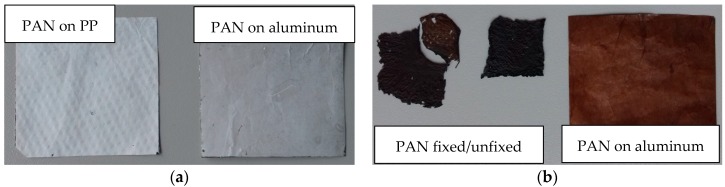
PAN nanofiber mats electrospun on different substrates: (**a**) after spinning; (**b**) after stabilization (and separating from the substrate in case of the PP substrates) at 280 °C for 1 h.

The same result can also be found on a macroscopic scale. As shown in Figure 3, the nanofiber mats electrospun on the usual PP substrate and on aluminum foil look very similar in their original state (Figure 3a). After stabilization at 280 °C for 1 h (Figure 3b), the PAN nanofiber mat on aluminum has the same dimensions as before. The unfixed pure PAN nanofiber mat has reduced its widths and lengths by approximately a factor of 2 each. The fixed pure PAN nanofiber mat was broken during stabilization; only the upper right corner remained under the weights, while the residual area shrank stronger. This significant dimensional change can also be recognized by the colors of the samples after stabilization—the brown color is much brighter for the PAN stabilized on aluminum, corresponding to less PAN per area due to the dimensional stability.

Such a strong adhesion between aluminum and polyacrylonitrile is usually not reported in the scientific literature. Aluminum oxide membranes, for example, can be used as a template to create PAN nanofibers by extrusion into this template and polymerizing in its nano-pores. In this case, the template can be recycled by washing, suggesting that the adhesion is not very strong [29,30]. Similarly, coating polyacrylonitrile and other polymers with aluminum resulted in condensation on the polymer substrate, forming a uniform metal layer there, while metals like copper and nickel migrated into the substrate [31]. Only by plasma polymerization of acrylonitrile gas is the adhesion of PAN layers on aluminum reported in the literature [32].

On the other hand, the solvent DMSO cannot contribute to the adhesion between the nanofiber mat and the aluminum substrate, either. Aluminum oxide (which forms a thin film on the surface of the uncleaned aluminum foil) is known to be stable in organic solvents, including DMSO [33,34,35,36].

**Figure 4:**
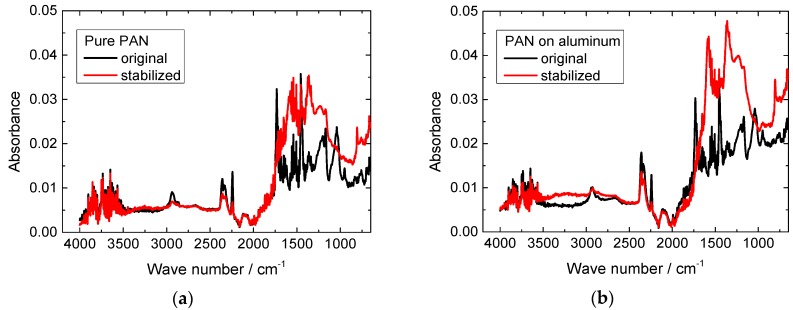
FTIR investigation of PAN nanofiber mats before and after stabilization: (**a**) pure PAN; (**b**) PAN on aluminum substrate.

Finally, one process which is sometimes mentioned in the literature may be responsible for the adhesion between both materials: for chemical vapor deposition coating of PAN with aluminum, the formation of aluminum carbide was observed [37], a reaction which is more typical for carbon/aluminum surfaces [38]. Another possible reaction—also typically only observed at high temperatures—is the formation of aluminum nitride [39]. In order to investigate whether any of these chemical reactions has occurred during electrospinning PAN on aluminum foil, FTIR investigations were performed. Exemplary results are depicted in Figure 4. The graphs show the typical peaks of pure and stabilized PAN in both cases, with an additionally increased absorbance for the smaller wavenumbers, as it can be expected from the aluminum oxide underground.

As described in detail by Mólnar et al. [15] and Sabantina et al. [27], PAN shows several characteristic peaks before stabilization, some of them from PAN and other from the different copolymers used to improve the properties of the fibers such as methyl acrylate. At 2938 cm^−1^ and 1452 cm^−1^ as well as 1380 cm^−1^, peaks correlated with bending and stretching vibrations of CH_2_ are visible. The peak at 2240 cm^−1^ is attributed to the stretching of nitrile functional group C≡N. The carbonyl (C=O) stretching peak can be recognized at 1732 cm^−1^. In the ranges of 1230–1250 cm^−1^ and 1050–1090 cm^−1^ peaks occur due to ester (C–O and C–O–C) vibrations of the co-monomers like itaconic acid or methyl acrylate.

After stabilization, these peaks attributed to nitrile and carbonyl functional groups are mostly vanished. Instead, C=N stretching vibrations at 1582 cm^−1^ and C=C stretching vibrations at 1660 cm^−1^ appear as a consequence of the cyclization–aromatization of the polymers. Additionally, the peak at 1360 cm^−1^ can be attributed to C–H bending and C–H_2_ wagging. Finally, the peak around 800 cm^−1^ is related to aromatic C–H vibrations due to oxidative dehydrogenation aromatization. As a consequence of the stabilization in oxidative atmosphere (air), oxygen cross-linking is formed between polymer chains and an increase in the absorbance can be observed in Figure 4 in the ranges of 1230–1250 cm^−1^ and 1050–1090 cm^−1^ attributed to C–O and C–O–C vibrations, in the case of the spectra of the stabilized fibers with respect to the electrospun ones.

Comparing PAN electrospun on aluminum with the pure PAN nanofiber mat electrospun on PP, no additional peaks are visible. Aluminum carbide should show peaks related to C–C and C=C in the range between 1350 and 1700 cm^−1^ which are not visible here [40]. This, however, is not sufficient to exclude the possibility that a thin layer of AlC is created along the interfacial surface. Aluminum nitride only shows a peak around 550 cm^−1^ and would thus not be visible in our FTIR instrument. The FTIR analysis cannot help in understanding the good adhesion between both materials in this test series.

**Figure 5:**
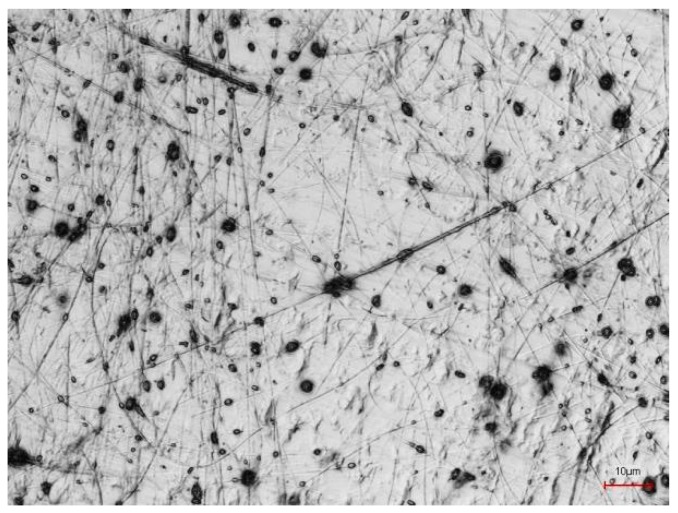
Aluminum surface after separating the PAN nanofiber mat electrospun on it. The scale bar indicates 10 μm.

Carefully separating the PAN nanofiber mat from the aluminum substrate shows that the adhesion between both materials is mostly of electrostatic nature, i.e., introduced by the electrospinning process itself. Nevertheless, the fibers at the interface are chemically bonded to the aluminum foil and cannot be separated without destroying the metal surface, as Figure 5 reveals. Apparently the adhesion between both materials can be attributed to a strong electrostatic interaction in combination with a chemical bonding of yet unknown nature.

**Figure 6:**
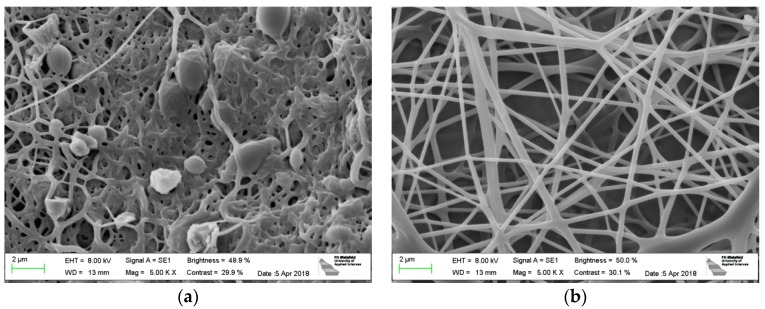
PAN nanofiber mats after carbonization at 500 °C: (**a**) electrospun on PP (substrate separated before stabilization); (**b**) electrospun on aluminum foil (substrate not removed before stabilization or carbonization).

Subsequent, the stabilized samples were carbonized at 500 °C (Figure 6). In both cases, the carbonized samples show morphologies very similar to those obtained after stabilization. Afterwards, it is possible to separate the nanofiber mat and aluminum foil with any scraping tool, if desired.

**Figure 7:**
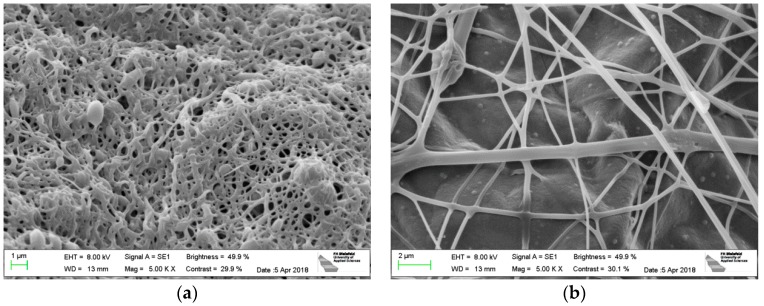
PAN nanofiber mats after carbonization at 800 °C: (**a**) electrospun on PP (substrate separated before stabilization); (**b**) electrospun on aluminum foil (substrate not removed before stabilization).

Finally, the stabilized PAN nanofiber mats were carbonized at 800 °C (Figure 7). Since this temperature is higher than the melting temperature of aluminum of approx. 660 °C, now both materials start intermixing and forming a composite, as can be recognized in Figure 7b. This technological approach of composite production is, to the best of our knowledge, not yet found in the scientific literature. 

A strong adhesion between aluminum and PAN has also been reported for other technologies in which both these materials were coated on each other in different ways. Carbon fibers were, e.g., coated with aluminum to create carbon/aluminum composites. Fibers along a fracture surface were not found to be pulled out of the aluminum composite, indicating a good adhesion [41]. When mixing short carbon fibers into liquid aluminum, their wettability was found poor [42]; thus, this new attempt may give rise to an interesting method to produce carbon/aluminum composites.

**Figure 8:**
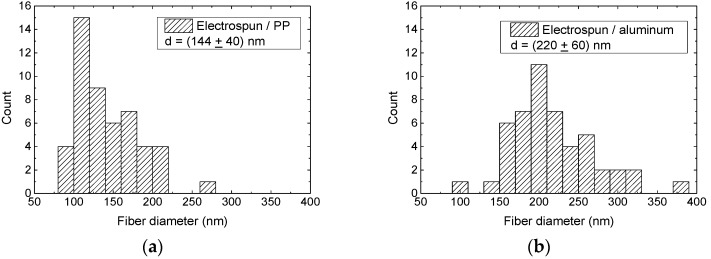

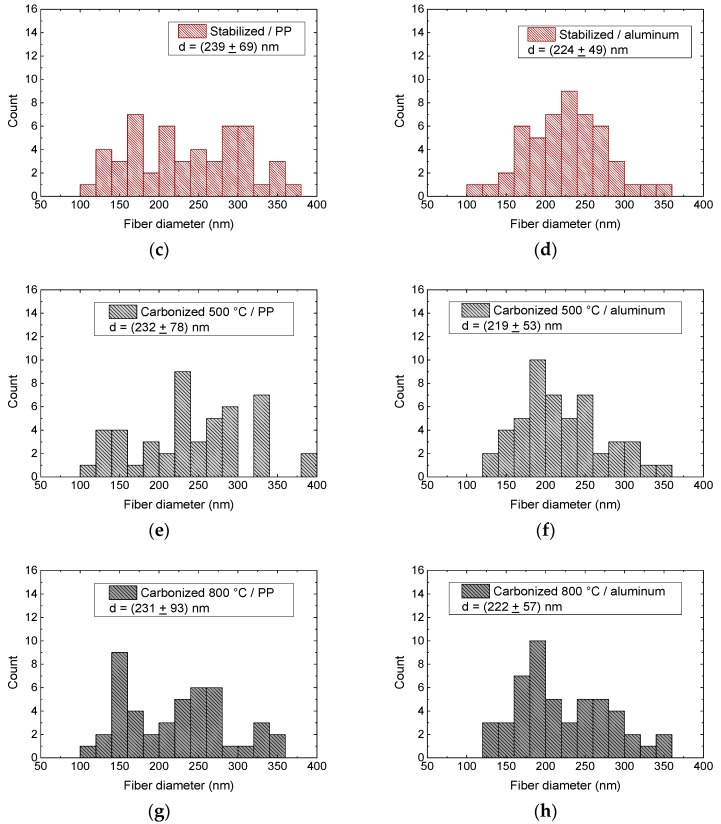
Diameters of PAN nanofiber mats electrospun on PP (**a**) or aluminum substrates (**b**), measured directly after spinning, after stabilization (**c**,**d**, respectively), and after carbonization at 500 °C (**e**,**f**) and 800 °C (**g**,**h**), respectively. Average diameters and their standard deviations are given in the insets.

To summarize the morphological changes during stabilization and carbonization, Figure 8 depicts a quantitative comparison of all nanofiber diameters, measured in the SEM images on 50 fibers per sample. 

As already visible from the SEM images themselves, the diameters of the electrospun PAN nanofibers on aluminum were significantly larger than those of the fibers spun on the PP nonwoven (Figure 8a,b). The stabilization of PAN fibers (prepared on PP substrate) produces an increase in the fiber diameter while the average nanofiber diameter on the aluminum foil stays constant (the undesired conglutinations, as visible from the SEM images, were not taken into account for calculation of this value, only clearly visible fibers) (Figure 8c,d). Carbonization at 500 °C (Figure 8e,f) or 800 °C (Figure 8g,h) does not change the average diameters anymore, neither for the nanofiber mats spun on PP nor for those stabilized and carbonized on aluminum. It should be mentioned that the standard deviation—i.e., the distribution of the nanofiber diameters—is slightly larger for the nanofibers prepared on the nonwoven PP after stabilization and carbonization.

## 4. Discussion

Electrospinning PAN nanofiber mats on aluminum substrates was shown to offer a simple possibility to overcome the problem of how to fix the nanofiber mats during the stabilization process, which is indispensable for keeping unconglutinated, straight nanofibers. Additionally, at higher carbonization temperatures, aluminum–carbon composites can be formed. 

It should be mentioned that in the study reported here, this composite formation was neither forced (e.g., by placing a weight on top during carbonization) nor investigated further since the main aim was finding a solution for the problem of undesired fiber shrinkage and bending during stabilization. Nevertheless, this effect results in many possible applications in the area of composites and will be investigated further in the near future, especially with respect to composite formation during carbonization and the mechanical properties of the resulting composites.

## 5. Patents

A patent is pending.
